# The Young Nephrologists’ Platform: the gateway to the future of nephrology

**DOI:** 10.1093/ckj/sfae024

**Published:** 2024-02-08

**Authors:** Orsolya Cseprekal, Amaryllis H Van Craenenbroeck, Martin H de Borst, Kate I Stevens, Ana Carina Ferreira, Miklos Z Molnar, Paulina Pettinelli, Andrzej Wiecek

**Affiliations:** Department of Surgery, Transplantation and Gastroenterology, Semmelweis University, Budapest, Hungary; Department of Nephrology, University Hospitals Leuven, Leuven, Belgium; Department of Internal Medicine, Division of Nephrology, University Medical Center Groningen, University of Groningen, Groningen, The Netherlands; Glasgow Renal and Transplant Unit, Queen Elizabeth University Hospital, Glasgow, UK; Department of Nephrology – Hospital Curry Cabral – ULS São José, Lisbon, Portugal; Nova Medical School, Lisbon, Portugal; Department of Internal Medicine, Division of Nephrology & Hypertension, Spencer Fox Eccles School of Medicine at the University of Utah, Salt Lake City, UT, USA; European Renal Association, Parma, Italy; Department of Nephrology, Transplantation and Internal Medicine, Medical University of Silesia, Katowice, Poland

The future is for young people and so in an increasingly hectic world, investment in the younger generations is essential—not just through education but by simultaneously supporting their dreams and aspirations. Recognizing this, in 2013, Prof. Andrzej Wiecek championed the concept of the Young Nephrologists’ Platform (YNP). A decade has now passed since the YNP was created and it is an appropriate time, therefore, to reflect upon the activities and achievements of the platform (Table 1).

**Table 1:** ERA YNP board since 2013.

Chair of YNP	Board members	Coordinator	ERA Presidents
Miklos Z. Molnar (2013–15)	Ana Carina Ferreira	Monica Fontana	Raymond Vanholder (2011–14)
	Davide Bolignano		Andrzej Wiecek (2014–17)
	Mariusz Kusztal		
	Maria Majernikova		
	Kultigin Turkmen		
	Giovana Seno Di Marco^c^		
	Julien Zuber^c^		
Ana Carina Ferreira (2015–17)	Miklos Z. Molnar (Past-Chair)^a^	Muguet Koobasi	Andrzej Wiecek (2014–17)
	Davide Bolignano		Carmine Zoccali (2017–20)
	Mariusz Kusztal^a^		
	Maria Majernikova^a^		
	Kultigin Turkmen^a^		
	Giovana Seno Di Marco^c^		
	Julien Zuber^c^		
	Rafael Kramann^a^,^c^		
	Kate Stevens^c^		
	Martin de Borst^b^,^c^		
	Emilie Cornec-Le Gall^b^,^c^		
	David Arroyo^b^		
Kate Stevens (2017–19)^c^	Ana Carina Ferreira (Past-Chair)^a^	Monica Fontana	Carmine Zoccali (2017–20)
	Martin de Borst^c^		
	Emilie Cornec-Le Gall^a^,^c^		
	David Arroyo		
	Davide Bolignano^a^		
	Maria Haller^b^		
	Abduzhappar Gaipov^b^		
	Albertien Van Eerde^b^,^c^		
	Shrikant Ramesh Mulay^c^		
	Thomas Schachtner^b^		
Martin de Borst (2019–21)^c^	Kate Stevens (Past-Chair)^a^,^c^	Monneth Briones	Carmine Zoccali (2017–20)
	Maria Haller^a^		Christoph Wanner (2020–24)
	Abduzhappar Gaipov^a^		
	Albertien Van Eerde^a^,^c^		
	Shrikant Ramesh Mulay^a^,^c^		
	Beatriz Sanchez-Alamo^b^		
	Amaryllis H. Van Craenenbroeck^b^		
	Rebecca Herzog^b^,^c^		
	Orsolya Cseprekal^b^		
	Andreas Kronbichler^b^		
	Ewa Pawlowicz^b^		
Amaryllis H. Van Craenenbroeck (2021–23)	Martin de Borst (Past-Chair)^a^,^c^	Monneth Briones/Paulina Pettinelli	Christoph Wanner (2020–24)
	Orsolya Cseprekal		
	Beatriz Sanchez-Alamo^a^		
	Rebecca Herzog^a^,^c^		
	Andreas Kronbichler^a^		
	Ewa Pawlowicz^a^		
	Hugo Diniz^b^		
	Safak Mirioglu^b^		
	Thimoteus Speer^b^,^c^		
	Lauren Floyd^b^		
	Marieta Theodorakopoulou^b^		
Orsolya Cseprekal (2023–25)	Amaryllis H. Van Craenenbroeck (Past-Chair)^a^	Paulina Pettinelli	Christoph Wanner (2020–24) Roser Torra (2024–27)
	Hugo Diniz		
	Safak Mirioglu		
	Lauren Floyd		
	Marieta Theodorakopoulou		
	Fernando Caravaca-Fontán^b^		
	Balazs Odler^b^		
	Elisabet Van Loon^b^		

a Leaving the board during the term.

^b^Starting the term.

^c^Stanley Shaldon winners: 2012 – Giovana Seno Di Marco, Germany; 2013 – Julien Zuber, France; 2014 – Rafael Kramann, Germany; 2015 – Kate Stevens, United Kingdom; 2016 – Emilie Cornec-Le Gall, France; 2017 – Albertien van Eerde, The Netherlands; 2018 – Shrikant Ramesh Mulay, United Kingdom; 2019 – Rebecca Herzog, Austria; 2020 – Martin De Borst, The Netherlands; 2021 – Thimoteus Speer, Germany.

**Figure:**
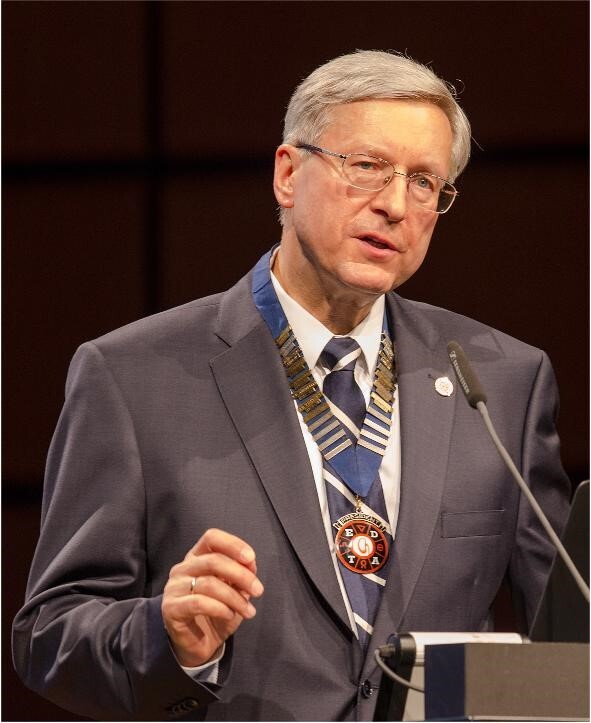
Andrzej Wiecek, Secretary/Treasurer (2011–14) and President (2014–17) of the ERA-EDTA

“In creating the YNP, my primary motivation was to involve young nephrologists in all activities of the European Renal Association–European Dialysis and Transplant Association (ERA-EDTA). The concept was initiated at the Prague Post-Congress Council Meeting in 2011. After extensive discussions, 18 months later in early 2013, the first YNP Chair was elected: Dr Miklos Z. Molnar from Hungary. The YNP board comprised five other members, representing the various geographic regions of Europe and ex-officio members—previous winners of the ERA-EDTA Young Nephrologist Award.

The mission of the YNP is to evaluate and understand the needs of young physicians and scientists in order to develop education, research, professional development and leadership

opportunities within the society, and specifically tailored to their needs. The YNP board created a blog to facilitate communication between board members and sent a letter to the Presidents of the National Societies with information about the aims and criteria of YNP membership [1].

During the 50th ERA-EDTA Congress in Istanbul in 2013, The ERA-EDTA Council was able to officially announce the creation of a body to better engage its talented younger members in shaping the future of the society—the YNP. The first logo of YNP was also presented (Fig. 1). The inaugural YNP congress session organized by the YNP board took place during this congress and was chaired by Dr Molnar.”

**Figure 1:**
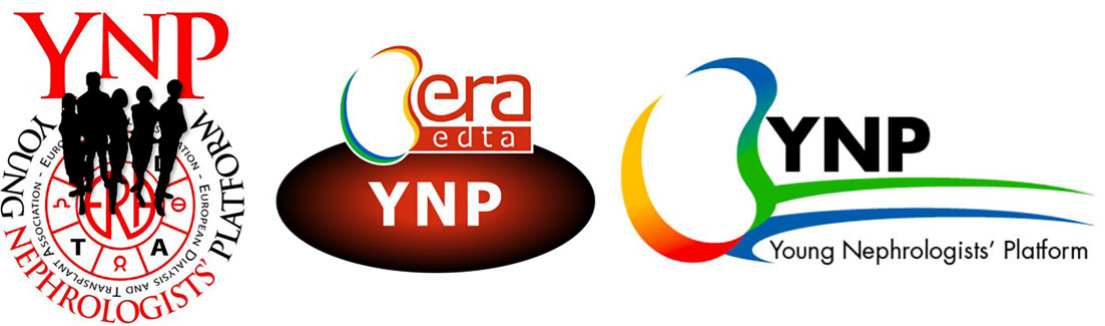
Left: the ERA-EDTA YNP logo from 2013. Middle: the ERA-EDTA YNP logo from 2018. Right: the present ERA YNP logo, introduced in September 2021.

**Figure:**
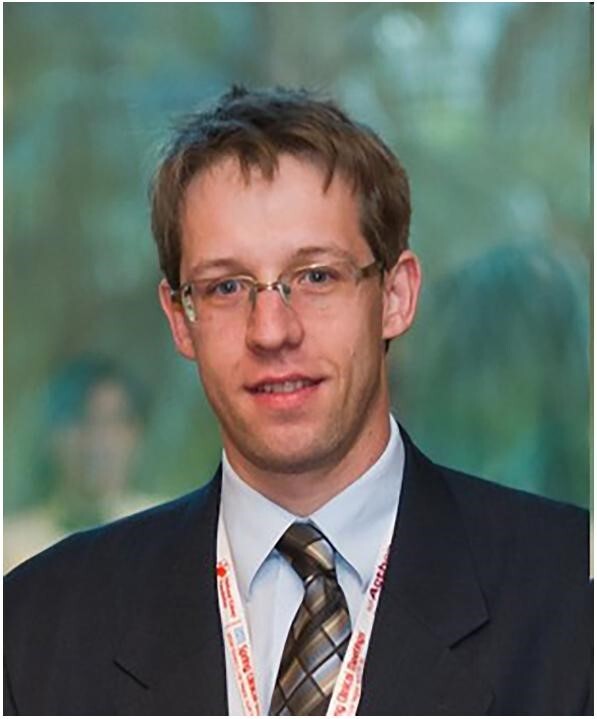
Miklos Z. Molnar, YNP Chair 2013–15

“I was lucky to be elected as the first Chair of YNP and served in this position between 2013 and 2015. With mixed feelings of fear and excitement, I set to work with the belief that this platform would allow radical change for young nephrologists in Europe. Looking back, I was very fortunate. I had unconditional support from Prof. Wiecek and the initial board was a great team of like-minded individuals who made the work straightforward and, honestly, much more fun than I had envisaged.

Initially, our focus was to publicize this new platform for younger nephrologists. We wanted to increase the membership, and we wanted to understand what people might expect from this platform. To achieve this, we reached out to our membership and asked about their needs. After analysing the responses, we established several programs. Many of these remain active ventures, 10 years later.

A free membership program for selected individuals was established along with an advisory program. Here young members, without a local advisor, could be matched with well-established ‘remote’ advisors, often based in other countries. At the beginning of the program, 19 advisors signed up and we were able to support six young nephrologists. I served as one advisor and I was connected with a young, keen nephrologist from Kazakhstan, who eventually worked with me for years. He published several papers, later became a YNP board member and is someone with whom I have established a lifelong friendship.

I learned a lot from YNP. I was faced with many new challenges: creating standard rules for an organization, budgeting, promotion of a new concept but I met these head on. The positive feedback and the friendships I established made it more than worthwhile and I reflect proudly on our achievements. At the end of my 2-year term, I was delighted that our first woman chair was elected.”

**Figure:**
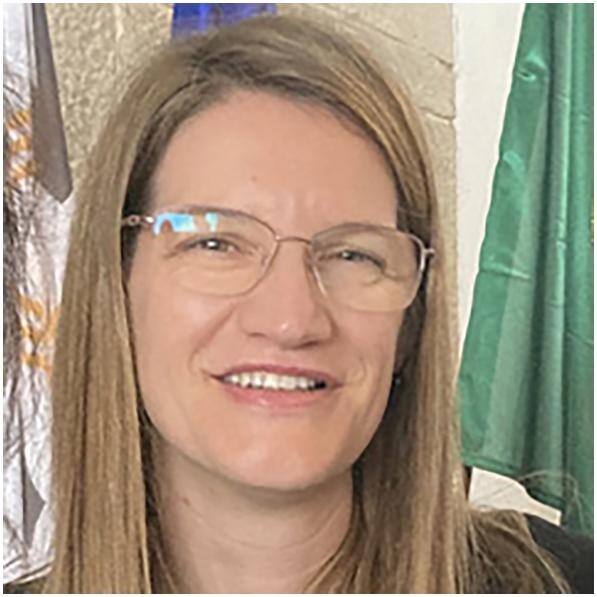
Ana Carina Ferreira, YNP Chair 2015–17

“In 2013, I was one of the founder members of the YNP along with our board members at that time. The members of the board increased with the inclusion of the Stanley Shaldon award winners from 2012 and 2013. All board members stayed for a minimum of 3 years but the six founder members stayed up to 5 years, according to age, to ensure continuity. Over the next 3 years, up until 2017, seven others became board members, three of whom had been Stanley Shaldon award winners (Table 1).

The number of members and the YNP influence has increased steadily. By 2012, there were around 900 members. By 2018, 1800 members were registered—an excellent achievement, aided by an opt-out YNP membership for all ERA-EDTA members up to 40 years.

Being YNP Chair between 2015 and 2017 was a privilege. One of the aims of the platform was that it should facilitate educational opportunities for young nephrologists, and to achieve this, we introduced a variety of successful initiatives.

One of the first was organization of an annual continuous medical education (CME) course held in different countries. The first course ‘Landmark studies in kidney transplantation’ was held in Budapest, Hungary in 2014. In 2015, ‘Glomerulonephritides’ took place in Istanbul, Turkey. Taken into account the success of these courses, in 2016, YNP held two courses: ‘Pregnancy and kidney disease’, in Lisbon, Portugal and ‘Interventional nephrology’ in Wroclaw, Poland. An ‘Acute kidney injury’ course was held in Dunblane, Scotland in 2017, and finally in 2018 ‘How to become your local expert in nephrogenetics’ was run in Belgrade, Serbia. These annual CME courses proved to be very popular and the low registration fees plus travel grants helped to make this educational opportunity very accessible.

During my term, YNP started the Advisory Program, with the aim of engaging young people with science and clinical nephrology. This was designed to pair YNP members with an expert senior in the field of nephrology. It is very reassuring to see that, after 10 years, it is still active, with minor changes (mentees and advisors can meet in person whereas previously it was all online).

‘Hot topics’ was also developed; this was a lot of fun. We reached out to experts in specific fields who created summaries of specific topics. We developed multiple choice questions (MCQs) based on these expert summaries. The summaries and MCQs were published on our website (and the NDT-Educational website) and allowed our colleagues to read the summaries and then test their new knowledge trying to answer the MCQs.

At the ERA-EDTA Congresses, YNP initially had a special session where the presenters were young, recognized investigators. However, the topics were very wide the sessions were not very well attended, likely as a result of the broad nature of them. We therefore decided instead to invite young speakers to present their work during the different sessions of the Congress, which provided much better visibility for the speakers and the YNP.

During my tenure, we initiated collaborations with other societies in nephrology including the European Society of Pediatric Nephrology (ESPN) and the American Society of Neprhology (ASN), but also outside of nephrology with the European Alliance of Associations for Rheumatology (EULAR).

From my point of view, although it was 2 years of intense work, I have been left with only positive memories.”

**Figure:**
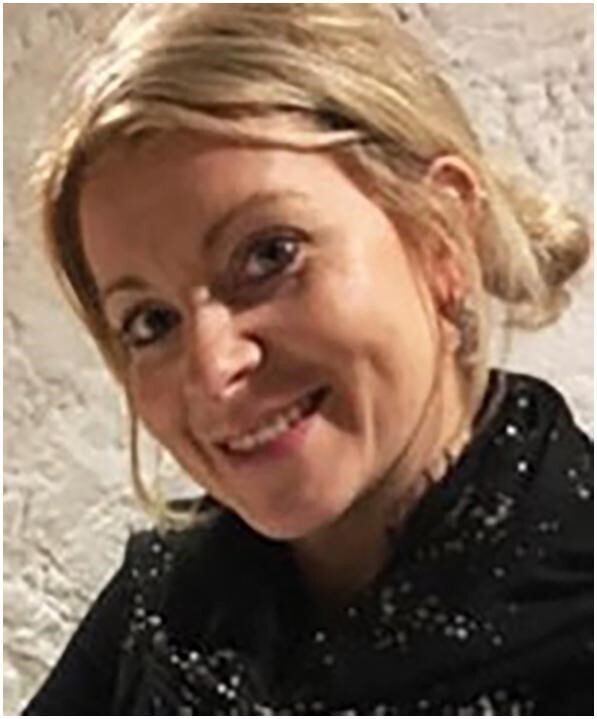
Kate Stevens, YNP Chair 2017–19

“Being honest, I joined YNP in 2015 by default, and not necessarily by choice. The platform remained fairly new, and I was unclear as to its remit. Reflecting now, I am delighted to have ‘accidentally’ joined the board and feel a great affection for the YNP and what it has achieved for the younger members of the ERA-EDTA community.

By 2015, YNP was really starting to come into its own in the main thanks to the hard work and dedication of the existing board but also because of the significant support from influential ERA-EDTA members who really got behind the platform. It was becoming increasingly apparent that the YNP was providing for the needs of younger nephrologists, where previously they had been overlooked (albeit unintentionally), and our greatest supporters were and are those who appreciate that youth is always the future, and the ERA must nurture the younger generations.

During my term, we focused on the main aims of YNP, established by our founding members: engaging young nephrologists in all ERA-EDTA activities, striving to support and promote the work of young nephrologists, and providing them with educational opportunities.

More specifically, these initiatives include the offer of free ERA-EDTA membership to the authors of the best abstracts submitted to the annual congress and free memberships to young authors publishing in *Nephrology Dialysis Transplantation* (*NDT*) or *Clinical Kidney Journal* (*CKJ*). The Advisory Programme continues to flourish and allows members to collaborate with experienced researchers and clinicians in specific areas of interest. The European certificate in Nephrology had its first sitting and our hot topics, established during Carina's term helped to provide a bank of practice questions, in the style of the exam, for our members. Additionally, we became active on social media, with support from the ERA-EDTA social media team.

We fostered strong links with *NDT* and *CKJ* and today continue to offer the opportunity to review articles to younger nephrologists who are looking to gain experience in this area. [2] Collaboration with other societies continued, including facilitating National Societies inviting YNP speakers to their local congresses. The aim was to increase visibility of younger nephrologists and to improve collaboration. The cost of attendance was split by YNP and The National Society. From 2018, we were able to offer ‘hands on’ interventional nephrology courses initially in partnership with The Vascular Access Society (VAS). These courses have flourished and currently run with the annual congress as an integral part of the CME courses.

The YNP ‘meet and greet’ had its inaugural event in Madrid in 2017 and since then it has become a well-established and highly anticipated social event for all YNP members. It allows networking and socializing in a relaxed environment and is funded by the ERA. The ‘meet and greet’ is a dedicated event for younger members of our community, some of whom may not always be included in other social activities (by virtue of being less established and well known) and for whom congresses can be prohibitively expensive.

Today, many of the original initiatives have developed and expanded such that younger nephrologists are not segregated but are included. Carina highlights YNP speakers included throughout the congress program. Other examples are YNP members now being represented on working groups, scientific committees and e-seminars, and in course and congress programs. This is the single most positive development and a real testament to the commitment of the YNP board and members together with the society to include young nephrologists and make them and their needs a priority.”

**Figure:**
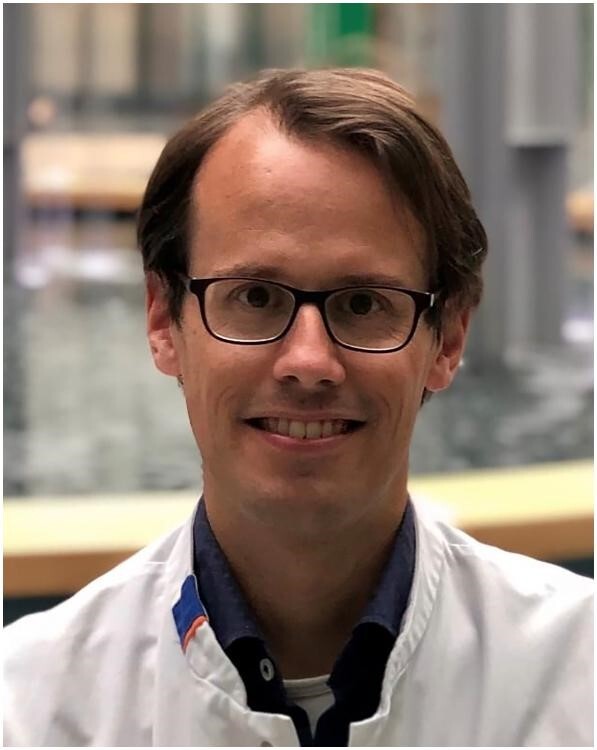
Martin de Borst, YNP Chair 2019–21

“Having been a YNP board member for 2 years, I was elected as the fourth chair starting at the 2019 Congress. No one could have anticipated what lay ahead of us at that time. The COVID-19 pandemic had a severe impact, not least on the lives of our patients. Subsequently, being on the front line of the fight against the pandemic, young European nephrologists faced unprecedented challenges. This shift of responsibilities, along with worldwide travel restrictions, drastically impacted YNP's physical activities in 2020. With most congresses restricted to online attendance, speaker exchange remained possible from behind the computer. Hands-on courses that had been very successful in previous years could not be continued live due to COVID-19 restrictions, but we managed to adapt and use virtual software.

We used the pandemic to focus our efforts on other activities that did not require physical presence. A relevant development, launched in 2021, was the YNP speakers’ database. This initiative was driven by a growing number of requests to provide excellent young speakers for the ERA (the organization's name was changed from ERA-EDTA to ERA in 2021) Congress and partner societies’ congresses. The YNP board was delighted by the increasing appreciation of the ERA Council and others that young nephrologists should be afforded a broader platform to share their excellent work. However, the identification of a diverse and high-quality team of young scientists turned out to be a challenging task. To better meet this challenge, the YNP speakers’ database was launched with the aim of composing a list of excellent young speakers that met at least one of the following criteria:

- have published a recent top publication;
- have generated a major breakthrough that impacts society;
- be considered to be within the best 10%–20% of their generation.

There were three ways for a YNP member to be included in this database: direct application, in response to an open call or via nomination by an ERA Council member, national representative or an ERA working group/committee/YNP board member. Upon approval by the YNP board, the speaker would enter the database, along with their topic of expertise. Following its launch, the concept of the YNP speaker database set the stage for an ERA-wide speaker database.

By June 2020, YNP had 1947 active members (57% women). One aspect we invested in during my term was the national representatives. We realized the importance of this given the size of YNP and the recognition that many countries already had national young nephrologist organizations with whom we could link. By 2021, we had 26 representatives covering the majority of European countries. Once in place, we invested much time and effort in keeping in touch with the representatives and hearing their voices during dedicated meetings at annual congresses. Not only did this allow YNP to better disseminate its activities, it also helped to gain input from active young colleagues within these countries regarding the content and organization of YNP activities.”

**Figure:**
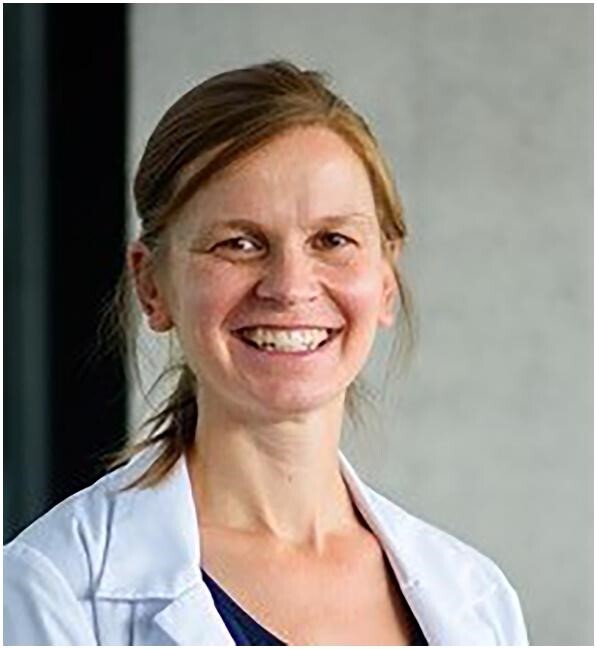
Amaryllis Van Craenenbroeck, YNP Chair 2021–23

“I took over the helm from Martin in 2021, in the midst of the COVID-19 pandemic with the reality of virtual meetings and challenging patient care. I was expecting my first child and looking forward to a bright future, a future which we could try to shape together. Together with Orsolya Cseprekal as Co-Chair and an ever-enthusiastic team of board members, we tried to get the most out of it. It was not the size of actions that mattered, but their impact.

Based upon this philosophy, we continued to build the significant progress made by the YNP and reached several milestones. One of our major accomplishments was getting the speakers’ database, conceived during Martin's term, actually up and running. This dynamic database has become an invaluable resource for the ERA community, offering a diverse range of experts and young key opinion leaders who can share their knowledge and insights.

Next, we successfully revived the mentorship program, which had been dormant. This program plays a crucial role in fostering professional growth and development among our members. By reinvigorating it, we provided a platform for experienced professionals to guide and support younger members, creating a nurturing environment for career advancement and skill development.

The YNP board members worked diligently to re-establish and strengthen our presence on social media platforms. By consistently sharing valuable content, engaging with our audience and promoting our events and initiatives, we gained visibility through modern channels used by young nephrologists (e.g. X). This allowed us to reach a broader audience and contributed to building a sense of community and belonging amongst our members.

Similarly, the new *CKJ* section ‘In Context’ [3] has also significantly enhanced the visibility and influence of the YNP community. This journal section features a distinctive approach where a YNP member collaborates with a well-established senior author to discuss the impact of a recent paper from a clinical perspective.”

**Figure:**
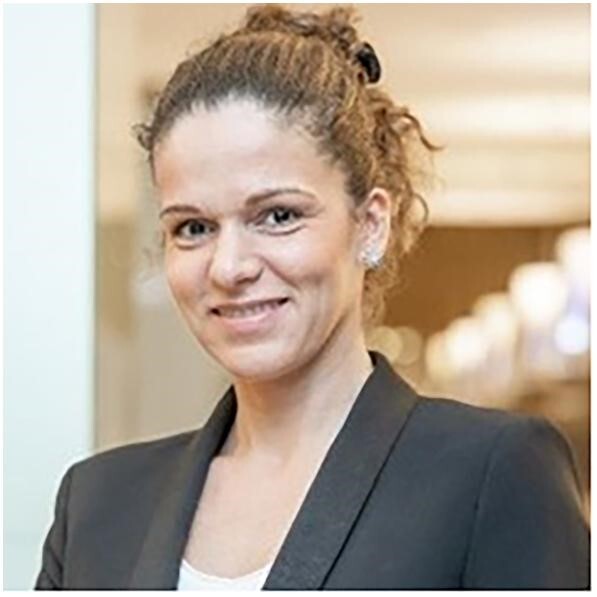
Orsolya Cseprekal, Chair 2023–25

“Significant headway has been made over the last decade (Table 1) and as the current Chair, together with the YNP board, our focus is to build upon these strong foundations and concentrate on community, networking, education and research opportunities for the younger generation of nephrologists.

There is a need to continue to reach out to the growing number of young nephrologists as well as to those who have not yet decided which branch of medicine they wish to pursue, thus ensuring ongoing visibility and accessibility of ERA and the YNP initiatives.

By 2023, YNP counted 4738 full and 807 associate members up to 40 years old from all over the world (62% female, 85% 29–40 years old, 15% below the age of 29 years; Fig. 2). Through national representatives, YNP also reaches those who may be a little further away from ERA's direct visual field.

**Figure 2:**
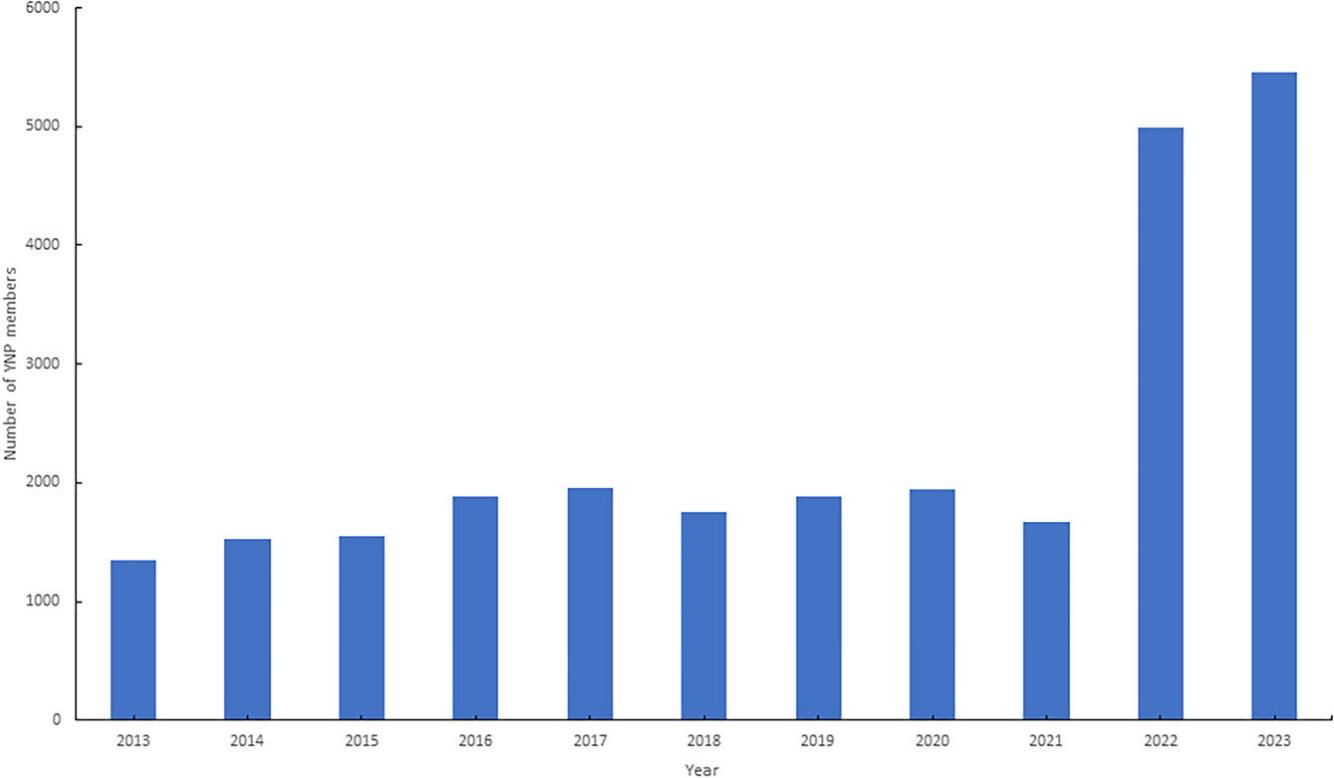
Absolute number of YNP members between 2013 and 2023. Note: Percentage of YNP members in ERA were 22% in 2013, 25% in 2014, 26% in 2015, 27% in 2016, 28% in 2017, 26% in 2018, 31% in 2019, 30% in 2020, 26% in 2021, 29% in 2022.

Social media activities have become indispensable, and it is with pleasure that YNP board members are included in the ERA Social Media team. This ensures ongoing visibility of YNP initiatives but also helps to bring younger people, who use these platforms, closer to ERA's programs. We are able to promote the latest research, guidelines and available opportunities within the ERA including working group positions and information about ERA fellowship grants.

By publishing regularly organized Q&A videos and the latest *NDT* or *CKJ* publications on our platforms, our followers are kept abreast of the latest key research developments. As an added benefit, social media helps to flatten the traditional hierarchy often seen in medicine and many authors themselves are directly accessible to our members via these platforms. Our interaction helps to bring readers and researchers together. YNP members continue to regularly review for both *NDT* and *CKJ* and also actively participate in editing the new educational tool from ERA: the ERA Neph-Manual.

We maintain a fruitful and active relationship with the American, Korean and Japanese Societies of Nephrology, organizing speaker exchanges and joint e-seminars annually. [4] Additionally, the European Society of Paediatric Nephrology (ESPN) and YNP joint meetings have taken place, and we eagerly anticipate the upcoming joint e-seminar between YNP and the young platform of the European Federation of Internal Medicine (EFIM). This will focus upon the interaction and overlap of internal medicine and nephrology and cover clinical and translational aspects.

The YNP board is strongly committed to helping the clinical and scientific careers of motivated young nephrologists and are delighted that our mentor–mentee program now counts 27 pairings since 2022. Thanks to the support of the ERA Council, the program has been broadened to include 1-week in person contact of the mentor and mentee.

One of the most important YNP tasks is to provide career role models for the younger generation. These role models should be at the forefront of clinical medicine, education and research. There must be particular emphasis on equality to ensure accessibility to all regardless of gender, race, career stage or family background. The ERA represents a community where it is possible to strike a balance between career and family and this should be publicized widely to the younger generation. It is possible to have it all.

In the years to come, there is little doubt that YNP will flourish as an integral part of the ERA community all over Europe and beyond. With invaluable support from the ERA Council, YNP will continue to support younger nephrologists, improve visibility for them and of nephrology as a specialty, and seek to meet the needs of its members whether that be with education, with networking or with simple career advice.

In the last decade, YNP has served as a golden gateway bringing younger members of the nephrology community together and alongside established members of the community. Ultimately, this has helped to shape the current era where the needs of the younger members are met, and age and experience no longer dominate.”

## References

[1] Zoccali C, Arici M, Blankestijn PJ et al. The ERA-EDTA today and tomorrow: a progress document by the ERA-EDTA Council. Nephrol Dial Transplant 2018;33:1077–82. 10.1093/ndt/gfy173 29796635

[2] Fouque D. Peer-reviewing and medical publication. Nephrol Dial Transplant 2022;37:1591–2. 10.1093/ndt/gfac183 35575615

[3] van Craenenbroeck A, Kronbichler A. Introduction to new CKJ section: In Context—in collaboration with YNP. Clin Kidney J 2023;16:1056 37398683 10.1093/ckj/sfad052 PMC10310497

[4] Wanner C. From WEDA to EDTA to ERA: 60 years of supporting European nephrology and counting. Clin Kidney J 2022;15:1439–46. 10.1093/ckj/sfac095 36824063 PMC9942442

